# Supplementary material for: End Sequence Analysis Toolkit (ESAT) expands the extractable information from single-cell RNA-seq data
Source: Genome Res. 2016 Oct;26(10):1397–410. doi: 10.1101/gr.207902.116 (PMC5052061; doi:10.1101/gr.207902.116)
Supplement: Supplemental Material [file supp_gr.207902.116_Supp_Materials.tar.gz › ESAT-esat_v0.1/doc/html/classbroad_1_1pda_1_1seq_1_1segmentation_1_1_genome_with_gaps2.html]

ESAT: broad.pda.seq.segmentation.GenomeWithGaps2 Class Reference


ESAT


- Main Page
- Related Pages
- Packages
- Classes
- Files

- Class List
- Class Index
- Class Hierarchy
- Class Members

All Classes Namespaces Files Functions Variables Enumerator Pages

- broad
- pda
- seq
- segmentation
- GenomeWithGaps2

Public Member Functions |
List of all members

broad.pda.seq.segmentation.GenomeWithGaps2 Class Reference

Collaboration diagram for broad.pda.seq.segmentation.GenomeWithGaps2:

[legend]

|  |  |
| --- | --- |
| Public Member Functions | |
|  | GenomeWithGaps2 (Collection< Annotation > gaps, String chr, int genomeSize, String orientation) |
|  | |
| Gene | getRelativeWindow (int relativeStart, int relativeEnd) |
|  | |
| int | getRelativeGenomeLength () |
|  | |

## Constructor & Destructor Documentation

|  |  |  |  |
| --- | --- | --- | --- |
| broad.pda.seq.segmentation.GenomeWithGaps2.GenomeWithGaps2 | ( | Collection< Annotation > | *gaps*, |
|  |  | String | *chr*, |
|  |  | int | *genomeSize*, |
|  |  | String | *orientation* |
|  | ) |  |  |

## Member Function Documentation

|  |  |  |  |  |
| --- | --- | --- | --- | --- |
| int broad.pda.seq.segmentation.GenomeWithGaps2.getRelativeGenomeLength | ( |  | ) |  |

|  |  |  |  |
| --- | --- | --- | --- |
| Gene broad.pda.seq.segmentation.GenomeWithGaps2.getRelativeWindow | ( | int | *relativeStart*, |
|  |  | int | *relativeEnd* |
|  | ) |  |  |

---

The documentation for this class was generated from the following file:

- java/broad/pda/seq/segmentation/GenomeWithGaps2.java


---

Generated on Fri Aug 15 2014 15:11:05 for ESAT by  

 1.8.7
